# On the Discovery of a New Fluid for Preserving the Colour of Pathological and Anatomical Preparations

**Published:** 1848-07

**Authors:** 


*On the Discovery of a new Fluid for preserving the Colour of pathological and anatomical Preparations.* By M. H. Stapleton, M. D., F. R. C. S., M. R. I. A., Surgeon to Jervis-street Hospital.

Sir,—I beg leave to make known to the profession, through the pages of your Journal the discovery of a solution, which I have employed during the last six years, for preserving pathological specimens. It possesses the important advantages of causing such preparations to retain their colour perfectly unchanged, and it does not harden the substance immersed in it. These two results are well instanced in several preparations in Jervis-street Hospital,—in particular, preparations of the brain, showing apolectic clot; apoplexy of the lung, and the recent appearances in stricture of the urethra.

The process I adopt is simply as follows:—In a quart of a saturated solution of alum in water I dissolve half a drachm of nitre; in this fluid I immerse the recent preparation, which soon becomes decolourized, but the colour gradually returns within a few days, the period, however, varying in different preparations. When the colour is thus completely restored, I put up the preparation in a filtered solution of alum. The specimens are open for inspection.

I remain your's, &c &c., M. H. STAPLETON

To the Editor of the Dublin Quarterly Journal, 16th Jan., 1848.

